# Hypertension Control Among Patients Receiving Care from a Field-Based Homeless Healthcare Team

**DOI:** 10.1007/s11606-026-10488-2

**Published:** 2026-06-25

**Authors:** Sae Takada, Madison Michelle Dawson, Un Young Rebecca Chung, Joshua Jaegun Lee, Catherine Weaver, Marjan Javanbakht, Evan Michael Shannon

**Affiliations:** 1 Division of General Internal Medicine and Health Services Research, David Geffen School of Medicine at UCLA, Los Angeles, CA USA; 2 University of California, Los Angeles, CA USA; 3 UCLA Health, Los Angeles, CA USA; 4 Department of Emergency Medicine, David Geffen School of Medicine at UCLA, Los Angeles, CA USA; 5 Department of Epidemiology, UCLA Fielding School of Public Health, Los Angeles, CA USA

## INTRODUCTION

People experiencing homelessness (PEH) face significant health disparities and elevated cardiovascular morbidity and mortality compared with the general population, in part due to disparities in hypertension control.^1,2^ Field-based homeless healthcare is a growing paradigm of care delivery for PEH in which preventive screenings are delivered by healthcare professionals on foot and via mobile vans equipped with medical supplies and pharmaceuticals, enabling teams to reach less accessible locations.^3^ There is limited evidence on whether field-based homeless healthcare improves the management of hypertension among PEH. This study examines whether PEH with hypertension receiving care from a field-based homeless healthcare program in Los Angeles County demonstrated improved blood pressure control.

## METHODS

### Setting

The UCLA Health Homeless Healthcare Collaborative (HHC) is a field-based homeless healthcare program that operates six mobile units staffed by clinicians, nurses, community health workers, and social workers across Los Angeles County. HHC provides primary care, urgent care, mental health care, and social services referrals to PEH at street encampments, shelters, motels, and pop-up events.

### Study Cohort

We extracted electronic health record (EHR) data for patients 18 or older who had encounters with HHC between January 2022 and November 2025. We included patients if they had a diagnosis of hypertension based on *International Classification of Diseases* (ICD)-10 diagnostic code (I10 Essential Hypertension), were prescribed anti-hypertensives, or had a systolic blood pressure ≥ 140 mmHg or diastolic blood pressure ≥ 90 mmHg, in accordance with Healthcare Effectiveness Data and Information Set (HEDIS) specifications,^4^ on two outpatient encounters. We included patients with at least three documented clinical encounters with HHC.

### Measures

We included numerical blood pressure values and coded blood pressure control (systolic blood pressure < 140 and diastolic blood pressure < 90) per HEDIS.^4^ We also extracted demographic variables (age, sex, race, and ethnicity).

### Statistical Analysis

Changes between the first and most recent blood pressure values recorded by HHC were compared using a paired *t*-test. McNemar’s exact test was used to compare blood pressure control between the first and most recent blood pressures.

## RESULTS

Among 887 adults in the hypertension cohort, the mean age was 55.1; 624 (70.4%) participants were male; 199 (22.4%) identified as Hispanic, 186 (21.0%) as non-Hispanic Black, 175 (19.7%) as non-Hispanic White, and 25 (2.8%) as Other; and 284 (32.0%) had missing race and ethnicity data (Table 1). The hypertension cohort had a median of five encounters with HHC (interquartile range (IQR) 4–9) and median follow-up time of 238 days between *T*_0_ and *T*_1_.

**Table 1:** Demographic and Clinical Characteristics of the Hypertension Cohort (*N* = 887)

Variable	Mean (SD) or *n* (%)
Age	
Mean	55.1 (12.0)
18–24	4 (0.5)
25–34	39 (4.4)
35–44	136 (15.3)
45–54	234 (26.4)
55–64	285 (32.1)
65–74	148 (16.7)
75–84	36 (4.1)
85+	5 (0.6)
Sex	
Male	624 (70.4)
Female	263 (29.7)
Race and ethnicity	
Hispanic all-races	199 (22.4)
Non-Hispanic Black	186 (21.0)
Non-Hispanic White	175 (19.7)
Other	25 (2.8)
Unknown	284 (32.0)

*SD* standard deviation

**Figure 1:**
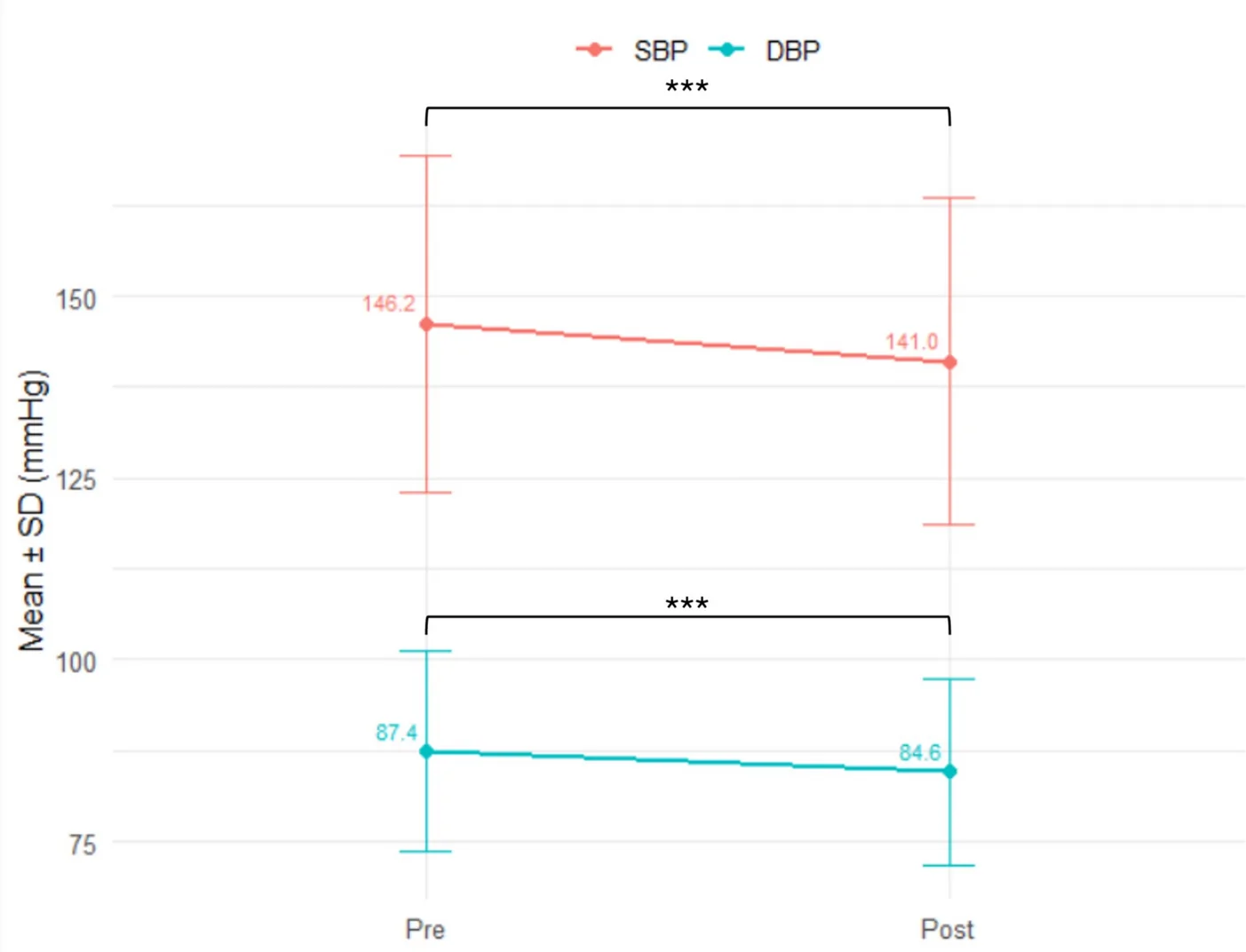
Blood pressure at baseline (pre) and most recent (post) values among Homeless Healthcare Collaborative (HHC) patients with hypertension, and overlying box and whisker plots. *** denotes *p* < 0.001. *P*-values derived from paired *t*-test. Abbreviations: DBP, diastolic blood pressure; mmHg, millimeters of mercury; SBP, systolic blood pressure.

Patients’ mean systolic blood pressure decreased from 146.2 mmHg (SD 23.1) at *T*_0_ to 141.0 mmHg (SD 22.5) at *T*_1_ (*p* < 0.001), while mean diastolic blood pressure decreased from 87.4 (SD 13.8) at *T*_0_ to 84.6 mmHg (SD 12.8) at *T*_1_ (*p* < 0.001) (Fig. 1). The number of patients with hypertension control increased from 308 (34.7%) at *T*_0_ to 402 (45.3%) at *T*_1_ (*p* < 0.001). Sensitivity analyses among patients prescribed anti-hypertensives showed similar results (results not shown).

## DISCUSSION

In this study of nearly 900 PEH with hypertension, at least 3 clinical visits, and a median of over 6 months of follow-up at a field-based homeless healthcare program in Los Angeles County, we found a significant and clinically meaningful^5^ decline in blood pressure between the first and most recent measures and a significant improvement in blood pressure control. Our findings provide preliminary evidence for the role of field-based homeless healthcare in improving chronic disease management for PEH.

Field-based homeless healthcare programs may effectively manage chronic diseases among PEH as they make a purposeful effort to remove barriers to healthcare, including bringing care to where they live, providing affordable, compassionate, and judgment-free care, and addressing competing needs. As field-based homeless healthcare expands across the country,^6^ further research is needed to ascertain the effectiveness of such teams in improving chronic disease control in this highly vulnerable population.

Study limitations include lack of patient data from other healthcare systems, lack of reliable data on underlying or new cardiovascular disease, self-selection bias among PEH who receive care at HHC, and lack of a dedicated comparison group to account for secular trends.

In conclusion, our findings demonstrate that field-based homeless healthcare programs have the potential to improve hypertension control among PEH and address the significant burden of cardiovascular disease faced by this population.

## Data Availability

As these data were derived from HIPAA-protected electronic medical records, they are not publicly available.
